# Assessment of ABCDE approach knowledge among residents and interns in multiple Egyptian hospitals, a cross-sectional study

**DOI:** 10.1186/s12909-025-06668-z

**Published:** 2025-01-31

**Authors:** Mohamed Saad Rakab, Ahmed B. Zaid, Malak Abbas Hamadein, Salma Abbas Hamadein, Mahmoud Abd Elgawad Ashour, Alaa Samir El-Shamia, Dina Ayman Mostafa, Rahma Mogahed Rateb, Marwa Abdelrahman Elashry, Mennat-Allah Mostafa El-Badawy, Rehab Shaheen Bahram Shaheen

**Affiliations:** 1 Faculty of Medicine, Mansoura University, Mansoura, Egypt; 2 Clinical Pathology Department, National Liver Institute, Menofia University, Shibin Al Kawm, Egypt; 3 Faculty of Medicine, Nile University, Abuja, Sudan; 4 Faculty of Medicine, Alexandria University, Bab Sharqi, Egypt; 5 Mansoura Manchester Medical Program, Mansoura, Egypt; 6 Faculty of Medicine, Benisuef University, Beni Suef, Egypt; 7 Faculty of medicine, Assuit University, Assiut, Egypt; 8 Mansoura University hospital, Mansoura, Egypt; 9 Alexandria University Main Hospital, Al Attarin, Egypt; 10 Internal medicine and critical care unit, Mansoura University, Mansoura, Egypt

**Keywords:** Emergency medicine, First aids, Medical residents, Medical interns, Egypt

## Abstract

**Background:**

The Airway, Breathing, Circulation, Disability, and Exposure (ABCDE) approach is crucial in emergency care, but there may be variability in adherence among healthcare professionals. Inconsistent application of this approach may lead to variations in patient care quality and outcomes. Identifying the factors influencing adherence can help improve training to ensure more effective application across emergency settings. This study explores the theoretical knowledge of the ABCDE approach among Egyptian resident doctors and medical interns.

**Methods:**

An online survey was conducted in Egypt targeting resident doctors and medical interns. Statistical analyses were performed using SPSS 26 and Excel, descriptive statistics and association tests were used to measure the relationship between knowledge and demographic factors.

**Results:**

The study included 422 medical residents and interns, with most in university hospitals. The average knowledge score of 59.1% exposed specific gaps in understanding, emphasizing deficiencies in 12 questions answered by less than 50%. Notably, 49.5% acquired ABCDE knowledge from medical school, while 28.2% had ALS/BLS courses. Encouragingly, 91.2% expressed willingness for life support training. Statistical analyses unveiled significant associations between knowledge scores and both medical practice settings and sources of ABCDE knowledge. Surgeons exhibited the lowest knowledge scores among participants, emphasizing the need for tailored interventions across specialties.

**Conclusion:**

This study addresses a critical gap in ABCDE approach knowledge among Egyptian resident doctors and medical interns. The study points to the need for focused education, especially for surgeons, to improve emergency care skills and patient outcomes through continued training.

## Introduction

The ABCDE (Airway, Breathing, Circulation, Disability, and Exposure) approach stands as a widely employed and universal method for evaluating and addressing critically unwell emergency patients. Professionals assert that employing the ABCDE approach enhances the timely assessment and treatment of individuals requiring emergency care (1,2). This systematic, step-by-step process facilitates efficient time management and early identification of deterioration, particularly crucial during the “golden hour” – the initial hour post-injury or illness onset when resuscitation efforts are most beneficial (3). Suitable for patients of any age, international guidelines recommend the use of this approach whenever there is suspicion of a serious illness or injury, irrespective of the underlying cause. This recommendation is based on the recognition that critical conditions share similarities in medical signs (4).

All health care professionals regardless the specialty have to know how to follow the ABCDE approach in all critically ill, deteriorating or severely injured patients in order to manage any unexpected life-threating problems before moving to the next step of medical assessment.

**Fig. 1:**
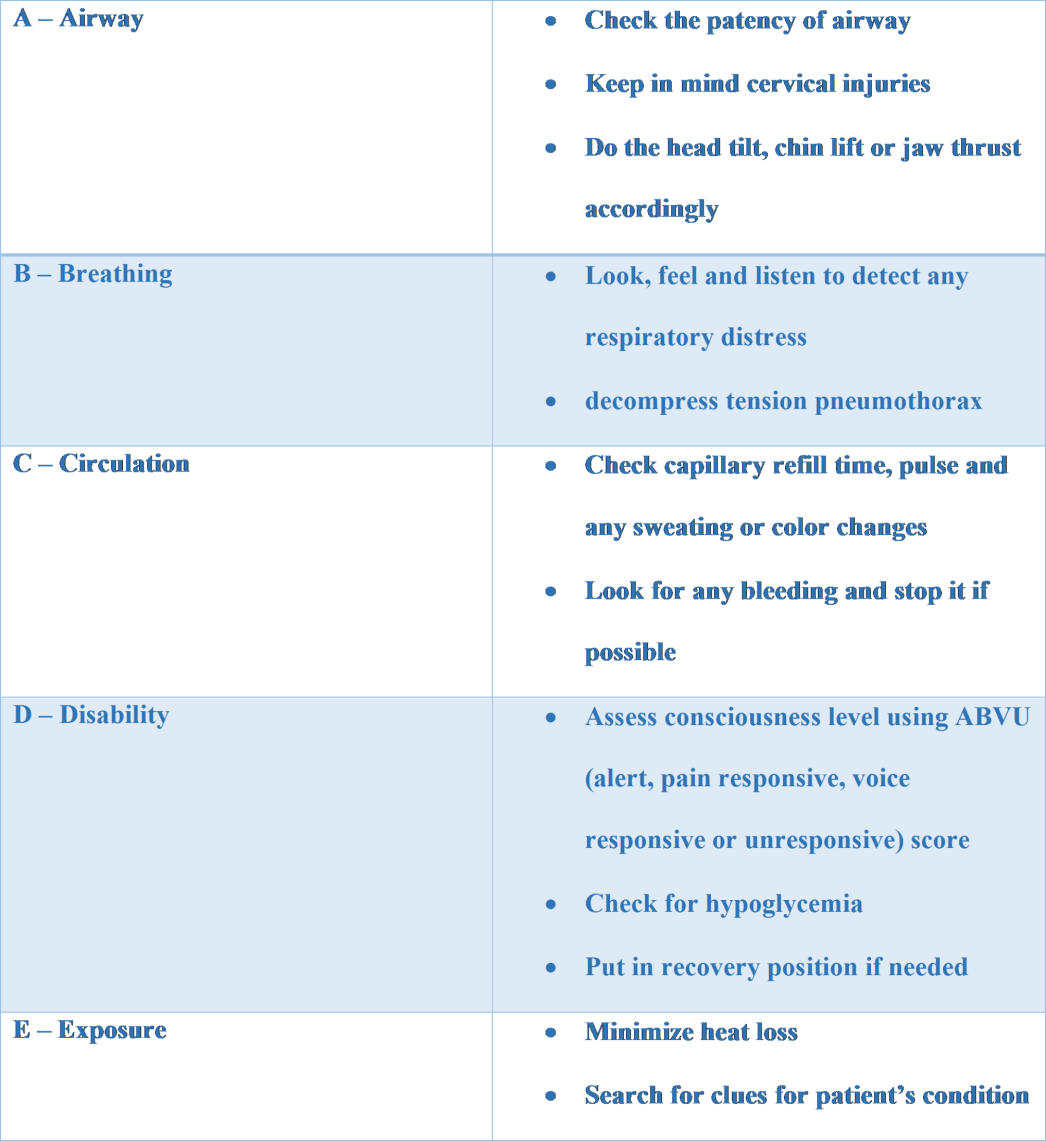
ABCDE algorithm

The critical points of ABCDE algorithm can be summarized in Fig. 1 [1]:

Healthcare professionals must apply their clinical judgement while focusing on the patient as a whole while using the ABCDE strategy. If done correctly, this brief yet thorough assessment can be completed successfully in less than five minutes [2].

Although the ABCDE approach is highly recommended, there are signs that practice has variable adherence among healthcare professionals [3]. A randomized clinical trial assessing the adherence to the ABCDE algorithm showed that the overall adherence to the approach was relatively low [4]. In Egypt, there is no study discussing the ABCDE approach knowledge level of health care professionals especially those with direct responsibility in emergency situations, residents and interns. As knowledge is fundamental for hospitality regarding life-saving approaches, assessment of ABCDE knowledge is needed.

## Methods

### Study design and setting

We conducted a cross-sectional study between June 7, 2023 and June 21, 2023 in various hospitals in Mansoura, Menofia, Alexandria, Benisuef, and Assuit governates in Egypt. This survey was based on a validated scale in a previously published study [3]. Data collection depended on convenience and snowball technique. A google form questionnaire was designed and distributed either by delivering the survey to potential respondents face to face or through multiple social media platforms. We clearly elaborated purposes of the study to the participants and stated that it was not obligatory to involve in the study. They were given clear instructions and worked under daily supervision by members of research team and the team leader.

### Study sample

The study’s inclusion criteria were Egyptian medical interns and resident doctors as these two groups more frequently face first aid-demanding situations in medical practice in Egypt. We excluded any uncompleted survey from the study. All participants agreed to enrol into the study with their own will without pressure and were provided with the identity of the research group, their right to discontinue the survey whenever they wanted, the right to data confidentiality, and that only completed valid submissions would be analysed. All these information was provided in the first page of the survey before answering the questions, and data was kept in a secure online database with access to research team only.

A single proportion of the population formula [n = [(Za/2)2P(1-P)]/d2] was applied to detect the minimal sample size needed for the study. With a 95% confidence level (Z a/2 = 1.96), a 5% margin of error, P = the estimated overall test score among resident doctors and medical interns based on previous literature (80%) (9) and adding 5% for a possible non-response rate, 246 interns and resident doctors were needed to proceed with this study. The final sample size was 422 interns and residents.

### Measures

The survey used in this study comprises two sections, with the first section focusing on sociodemographic data including medical role, specialty, place of medical practice, previous source of knowledge of ABCDE approach and the readiness to participate in life support training in free time, and the second section contained 29 multiple-choices questions assessing the ABCDE approach knowledge, developed and validated by Nino H. C. Schoeber et al. [3] Each question of the test has multiple possible choices, of which only one answer is correct. Each correct answer is considered one point, with a maximum score of 29 points for each participant. To make it simpler to understand the findings, the original 29-point scale was condensed to a 10-point scale. Any respondent scoring below 7.5 points was presumed to have not attained the required degree of theoretical understanding of the ABCDE approach, as those scoring 7.5/10.0 or more were deemed knowledgeable about the ABCDE technique.

### Ethical consideration

This study was undertaken after the approval of the Mansoura Faculty of Medicine IRB (R.23.03.2101). Respondents received a URL for a Google’s online survey via social media, mainly depending on Whatsapp or email and were asked if they consent to complete the survey before the questions starts to show up. When collecting the data face-to-face, the data collector had clear verbal consent from the respondent to participate. All participants were assured that the gathered information would not be used elsewhere but this study. Informed consent for publication was obtained from each participant. Confidentiality and anonymity were maintained in all steps of the study, and all the received data was saved in an online protected database.

### Statistical analysis

Statistical analysis was conducted using SPSS version 26 and Microsoft Excel to derive meaningful insights from the collected data. Descriptive statistics and frequencies were employed to categorize and present sociodemographic characteristics, including medical role, specialty, place of medical practice, previous source of ABCDE approach knowledge, and willingness to participate in life support training during free time. Knowledge levels were classified into “satisfactory” and “unsatisfactory” categories, determined by adjusted cutoff thresholds (above 75% and below 75% of the total score, respectively). To identify any statistical differences in knowledge toward the ABCDE approach, a Test de Kruskal-Wallis was performed, considering a p-value less than 0.05 as statistically significant. This test was specifically applied to assess the association between different subgroups and test scores.

## Results

### Demographic characteristics

We approached 450 medical interns and residents, of which, 422 completed and returned the questionnaire with a response rate of 93.8% with no missing data. The respondents were nearly distributed equally among 220 (52.1%) residents and 202 (47.9%) interns. In terms of settings of medical practice, University hospitals formed the majority 289 (68.5%) followed by public hospitals 107 (25.4%) and lastly, private practice 26 (6.2%). More details on the demographic and professional characteristics of the participants are provided in Table 1.

**Table 1:** Demographic and characteristics of the participants

Characteristics		Frequency	%
**Medical role**			
	Intern	202	47.9
	Resident	220	52.1
***Setting of medical practice***			
	University hospitals	289	68.5
	Public hospitals	107	25.4
	Private practice	26	6.2
***Specialty (for residents)***			
	Internal medicine	86	20.4
	Surgery	42	10
	Pediatrics	19	4.5
	Emergency medicine	18	4.3
	Family medicine	13	3.1
	Others	42	10

### Knowledge scores

The average score was 59.1% with mean and standard deviation of (5.91, 1.529) respectively. From the 29 questions asked, 12 were correctly answered by less than 50% of the participants. The details of questions and answers are demonstrated in Table 2.

**Table 2:** Questions of the ABCDE approach test

Question		Correct *n*(%)	Wrong *n*(%)
***What is usually a late sign of circulatory failure?***		153 (36.3)	269 (63.7)
***Where is the airway obstruction located when you hear expiratory wheezing?***		197 (46.7)	225 (53.3)
***At which locations can an internal bleeding cause a hypovolemic shock?***		339 (80.3)	83 (19.7)
***Which of the following aspects helps with assessing the effort of breathing***,*** additional to frequency of breathing?***		341 (80.8)	81 (19.2)
***What blood value is always part of the primary survey?***		194 (46)	228 (54)
***A patient has a capillary refill time of 4 s on the sternum. This is…***		322 (76.3)	100 (23.7)
***On which of the following moments in the assessment of the patient do you use the ABCDE approach?***		275 (65.2)	147 (34.8)
***What does the P in the AVPU-score mean?***		360 (85.3)	62 (14.7)
***Classify the items below in order of priority according to the ABCDE approach***: ***1. AVPU-score*** ***2. Assessing capillary refill time*** ***3. Look-listen-feel***		276 (65.4)	146 (34.6)
***What aspect is not part of the D in the ABCDE approach?***		192 (45.5)	230 (54.5)
***What is***,*** at any age***,*** most informative for determining an open airway?***		296 (70.1)	126 (29.9)
***Which of the following actions does not contribute to diagnosing a tension pneumothorax?***		271 (64.2)	151 (35.8)
***Which C-problem is treated prior to the actual ABCDE approach?***		221 (52.4)	201 (47.6)
***What are the 4 most essential parts for assessing a patient’s circulatory condition?***		188 (44.5)	234 (55.5)
***Which of the following aspects is assessed in the E of the ABCDE approach?***		169 (40)	253 (60)
***Which of the following parameters is most reliable when assessing the effectiveness of breathing (in ambient air)?***		248 (58.8)	174 (41.2)
***What is the most uniform sign of increased effort of breathing***,*** for all ages?***		118 (28)	304 (72)
***Upon what is the fixed order of the ABCDE approach mostly based?***		315 (74.6)	107 (25.4)
***Which item is assessed in the E of the ABCDE approach?***		191 (45.3)	231 (54.7)
***What is not a sign of shock?***		376 (89.1)	46 (10.9)
***A prolonged capillary refill time could be present in*** ***which of the following situations?*** ***1. Hypertension*** ***2. Hypothermia*** ***3. Shock***		369 (87.4)	53 (12.6)
***Which abnormality in a patient’s posture is most severe?***		119 (28.2)	303 (71.8)
***What is the safest maneuver to secure the airway of a trauma patient?***		160 (37.9)	262 (62.1)
***Which aspects of the pupils always have to be assessed for every critically ill patient?***		335 (79.4)	87 (20.6)
***What is the look-listen-feel method namely meant for?***		236 (55.9)	186 (44.1)
***Which muscle is an accessory breathing muscle?***		145 (34.4)	277 (65.6)
***What does a stridor (inspiratory) indicate?***		289 (68.5)	133 (31.5)
***What does jugular venous distension indicate?*** ***1. Anaphylaxis*** ***2. Heart failure*** ***3. Pericardial tamponade*** ***4. Pneumothorax***		345 (81.8)	77 (18.2)
***With what can you make a quick estimation of a patient’s awareness?***		192 (45.5)	230 (54.5)

Nearly half of the participants received their ABCDE approach knowledge only from curriculum received in medical school (49.5%). Only 28.2% had previous Advanced Life Support/ Basic Life Support (ALS/BLS) courses and 6.4% have received no knowledge at all. On the other hand, 91.2% showed their willingness to participate in life support training program if freely available.

### Associations with demographic factors

In this study, the Kruskal-Wallis test was employed to assess differences in ABCDE knowledge scores across various demographic groups, revealing a statistically significant association between setting of medical practice and test scores. Specifically, those in public hospitals had the highest mean scores (6.07), while those in private practice had the lowest (5.00). This may imply that public hospital environments provide more exposure to emergency situations or better access to relevant training. Those who participated in ALS/BLS courses had a lower mean score than those who received their ABCDE approach knowledge from medical school curriculum and those who received their knowledge from training of department. This association was also statistically significant. Medical role and specialty showed no association of importance to ABCDE approach knowledge noting that surgeons had the lowest scores among the participants. Details are shown in Table 3.

**Table 3:** Association between participants’ demographics and scores

		Score, mean (SD)	*P*-value
***Medical role***			**0.466**
	Resident	5.93 (1.62)	
	Intern	5.90 (1.43)	
***Setting of medical practice***			**0.025**
	Public hospitals	6.07 (1.41)	
	University hospitals	5.93 (1.52)	
	Private practice	5.00 (1.87)	
***Specialty (for residents)***			**0.247**
	Pediatrics	6.45 (1.13)	
	Family medicine	6.34 (1.47)	
	Emergency medicine	6.28 (2.17)	
	Internal medicine	5.90 (1.70)	
	Others	5.76 (1.31)	
	Surgery	5.59 (1.68)	
***Source of previous ABCDE approach knowledge***			**< 0.001**
	Curriculum received in medical school	6.41 (1.55)	
	Training by department	5.86 (1.42)	
	ALS/BLS courses	5.83 (1.39)	
	No knowledge received	4.38 (1.61)	

## Discussion

To our knowledge, this study is the first to explore the theoretical understanding of the ABCDE technique among residents and interns in Egypt, considering these groups are frequently involved in urgent medical emergencies. Despite the strong recommendation of the ABCDE approach, indications suggest varying adherence among healthcare professionals. A previous research in the Netherlands has directly examined theoretical knowledge about the ABCDE technique across different fields, breaking new ground in this aspect [3]. While prior studies have assessed knowledge of the primary survey, they often focused on life support courses rather than the ABCDE approach specifically [5, 6]. Notably, there is a gap in research regarding healthcare workers’ awareness of the ABCDE strategy in Egypt, especially among interns and residents who bear direct responsibilities in emergency situations. Evaluating ABCDE knowledge becomes imperative as proficiency in life-saving techniques is crucial, particularly within the hospitality industry.

Our study revealed a mean knowledge score of 5.91 and a standard deviation of 1.529, stood at 59.1%. This aligns with findings by Binkhorst and his team [7], who noted low retention of Pediatric Basic Life Support (PBLS) abilities among pediatricians and residents during advanced life support scenarios. The inadequate acquisition or retention of knowledge, crucial for adhering to the ABCDE algorithm, may partially explain incomplete or improper implementation [8, 9]. Our knowledge score appears to be 21% lower than Schoeber and his colleagues [3] theoretical test-based findings, and 24% lower than Olgers and his team [10] hands-on assessment, suggesting wide room for improvement.

Analysis of our survey data, comprising 29 questions, revealed that fewer than half of the participants answered at least questions correctly. This outcome underscores a significant gap in the participants’ understanding of the subject matter, suggesting areas of potential improvement in their knowledge of the ABCDE approach. The identification of specific questions that posed challenges for participants could provide valuable insights for targeted educational interventions and curriculum enhancements.

Moreover, our study disclosed that only 28.2% of participants had undergone prior ALS/BLS courses, despite 91.2% expressing openness to participating in a life support training program if accessible. While existing research supports the notion that life support courses enhance theoretical knowledge and practical abilities, our findings underscore the necessity of regular practice. Knowledge and skills tend to deteriorate within 3–6 months without consistent rehearsal according to previous studies [7, 11].

Surprisingly, participants with prior ALS/BLS courses had lower mean scores compared to those who learned about the ABCDE approach through medical school curricula or departmental training. Therefore, our study suggests that the mode of knowledge acquisition may influence the depth of understanding, warranting further exploration into the effectiveness of different educational approaches. In a previous randomized control trial, two different educational methods were assessed. Results of this study suggested that compared to the lecture group, the video-based education group adhered to the ABCDE strategy more closely (4). Such observation may be complementary to our findings in future studies.

A statistically significant correlation emerged between participants’ test scores and the environment of their medical practice, with public hospitals scoring the highest and private practices the lowest. Interestingly, this contrasts with Schoeber and his team’s findings, emphasizes the complexity of linking theoretical knowledge test scores to clinical practice adherence [3]. Setting a cut score for passing or failing our assessment scale proved challenging, given the difficulty in determining the ideal level of ABCDE approach understanding [12, 13]. While our test served as a threshold-free formative assessment, it illuminated a concerning lack of comprehensive understanding among healthcare workers caring for critically ill patients, as indicated by a wide range of test scores and substantial standard deviations.

Despite the absence of an association between test scores and medical role or specialization in our results, but it was worth noting that surgeons scored the lowest among all participants, warranting further exploration into specialty-specific training needs.

### Strengths and limitations

One of the key strengths of this study is its focus on a critical yet understudied area in emergency care, the knowledge of the ABCDE approach among Egyptian residents and interns, who are often the first responders in urgent medical situations. The large, geographically diverse sample size from various hospitals across Egypt enhances the generalizability of the findings. The use of a validated survey tool adds reliability to the data, while the combination of both face-to-face and online data collection ensured a high response rate (93.8%). Moreover, the study’s identification of specific gaps in ABCDE knowledge and the significant associations between knowledge levels and practice settings provide valuable insights for improving emergency training programs and developing more targeted educational interventions.

The exclusive focus on knowledge, without incorporating an exploration of the practical application of the ABCDE approach in real-life scenarios, is a notable limitation. Future research should strive for a more comprehensive evaluation that includes both theoretical and practical assessments. Another limitation stems from the reliance on online-reported data, particularly concerning participants’ sources of ABCDE knowledge and their willingness to engage in life support training. This introduces the potential for recall bias, as participants may not accurately remember or report their educational sources or intentions, affecting the reliability of these findings. Also, generalizability of the findings may be affected by convenience and snowball sampling methods and variations in medical education systems and healthcare practices in different healthcare entities in Egypt. The cross-sectional nature of the study limits the ability to assess knowledge retention or changes over time. A longitudinal study with follow-up assessments could provide insights into the persistence of ABCDE knowledge and the impact of ongoing medical education initiatives.

The study reported lower mean scores for participants who had undergone ALS/BLS courses without delving into the specific content, quality, or duration of these courses. Such limitation raised from unexpectedness of such finding along with concerns regarding recall bias, pre and post interventional assessment would provide a better solution in determining the educational deficit. Lastly, participants may have provided responses perceived as socially desirable, particularly when expressing their willingness to participate in life support training. This potential social desirability bias could impact the accuracy of reported attitudes and intentions.

Acknowledging these limitations is essential for a nuanced interpretation of the study’s findings and for guiding future research efforts to address these constraints for a more robust understanding of ABCDE approach knowledge and application.

## Conclusion

The average ABCDE approach knowledge score among medical interns and residents was low revealing specific gaps in understanding. These findings collectively suggest opportunities for targeted educational interventions, particularly in addressing specific knowledge gaps identified in the ABCDE approach. Future initiatives should consider tailoring training programs to different medical settings and exploring innovative methods to enhance the overall proficiency of medical interns and residents in life support skills.

## Data Availability

The analyzed data with the corresponding author is available upon receiving a responsible order.

## Abbreviations

ABCDE: Airway, Breathing, Circulation, Disability, Exposure
ALS: Advanced Life Support
BLS: Basic Life Support
PBLS: Pediatric Basic Life Support
AVPU: Alert, Voice, Pain, Unresponsive
SPSS: Statistical Package for the Social Sciences
IRB: Institutional Review Board
